# On the Use of Simple Geometric Descriptors Provided by RGB-D Sensors for Re-Identification

**DOI:** 10.3390/s130708222

**Published:** 2013-06-27

**Authors:** Javier Lorenzo-Navarro, Modesto Castrillón-Santana, Daniel Hernández-Sosa

**Affiliations:** SIANI, Universidad de Las Palmas de Gran Canaria, Campus de Tafira, Las Palmas de Gran Canaria 35017, Spain; E-Mails: mcastrillon@iusiani.ulpgc.es (M.C.-S.); dhernandez@iusiani.ulpgc.es (D.H.-S.)

**Keywords:** re-identification, surveillance, RGB-D, depth

## Abstract

The re-identification problem has been commonly accomplished using appearance features based on salient points and color information. In this paper, we focus on the possibilities that simple geometric features obtained from depth images captured with RGB-D cameras may offer for the task, particularly working under severe illumination conditions. The results achieved for different sets of simple geometric features extracted in a top-view setup seem to provide useful descriptors for the re-identification task, which can be integrated in an ambient intelligent environment as part of a sensor network.

## 1. Introduction

There has been an enormous development in camera-based systems in the last fifteen years. The management of the resulting large amount of acquired data justifies the development of automatic techniques to leverage the human operator monitoring overload, *i.e.*, the surveillance system costs. Another emerging application context where this kind of technology is playing an important role is in ambient intelligence scenarios. In this field, information from multiple networked sensors is fused into the system to assist in monitoring and decision-making tasks, including medical applications [1] and 3D semantic modeling [2].

Current human monitoring applications focus on non-overlapping camera networks to perform behavior analysis and automatic event detection. Thus, people detection and tracking approaches are required abilities to be applied in this context aiming at developing automatic visual surveillance systems [3].

The general computer vision re-identification problem refers to determining whether a person of interest has been previously observed by the system [4–6]. Recent literature about the problem of re-identification is mostly focused on appearance-based models. Among the appearance cues used for this problem, interest points, structural information and color have deserved researchers attention, so far [5,7,8]. Those works prove that 2D visual descriptors extracted from RGB images are a valid data source to solve, at least partially, the problem. In this sense, facial and clothing appearance information have already been used to re-identify individuals in photo collections and TV video [9]. However, the face pattern presents low resolution in most surveillance scenarios. Clothing descriptors alone are certainly weak, but can help to locate people with similar appearance within a limited period of time, which may be later confirmed by a human. Indeed, human beings employ external features, such as body contours, hair, clothes, *etc.*, to complement person description and improve identification, particularly in low resolution images [10].

The recent appearance of the Kinect sensor provides additional and affordable rough depth information coupled with visual images, offering sufficient accuracy and resolution for indoor applications [11]. Due to this fact, this sensor has already been successfully applied to detect individuals and estimate their body pose [12,13]. As stated by Harville [14], depth devices: (1) are almost insensitive to shadows and illumination changes; (2) provide additional 3D shape information; (3) include occlusion data; (4) add new types of features to the feature space; and (5) add a disambiguating dimension.

Those advantages have led to the integration of RGB-D sensors for re-identification purposes [15–18]. In [15], a previous camera calibration step is needed to build height maps, which allow the system to define body prints. Each body print summarizes the color appearance at different heights. A more recent implementation [17] makes use of a cylindrical representation. A signature is extracted from the skeleton in [16], computing geometric features that may be related to soft-biometrics. Satta *et al.*[18] proposed a multi-camera system for re-identification based on joint relative positions extracted from the skeleton provided by the Kinect SDK. They also developed a demonstrator that is able to process images from a pair of Kinect sensors providing frontal and back views.

Not one of those works has considered re-identification from a zenithal camera. Most of them extract features observing the whole body in optimal illumination conditions and mainly use the depth cue to ease the segmentation module. However, different authors state the implicit advantage of using depth information to reduce certain ambiguous situations. In this sense, the use of stereo pair based approaches [19] has been proposed to take advantage of the depth information, reducing the inherent illumination problems. Certainly, their performance is still affected by bad or changing illumination conditions, as the correspondence map is based on visual information.

Top view cameras have already been used in surveillance applications [20], avoiding, in many cases, the need of an accurate calibration step. The top view configuration has the advantage of being privacy preserving, because the face is never recorded by the camera. However, depth information provides new features that are easy to extract. They lack the distinctiveness to identify an individual uniquely, but provide some evidence that can be used to support or discard a given hypothesis. In the experimental setup, the objective is not to identify precisely any identity, but to assist human operators in locating similar individual(s), as assumed by the re-identification literature.

**Figure 1.**
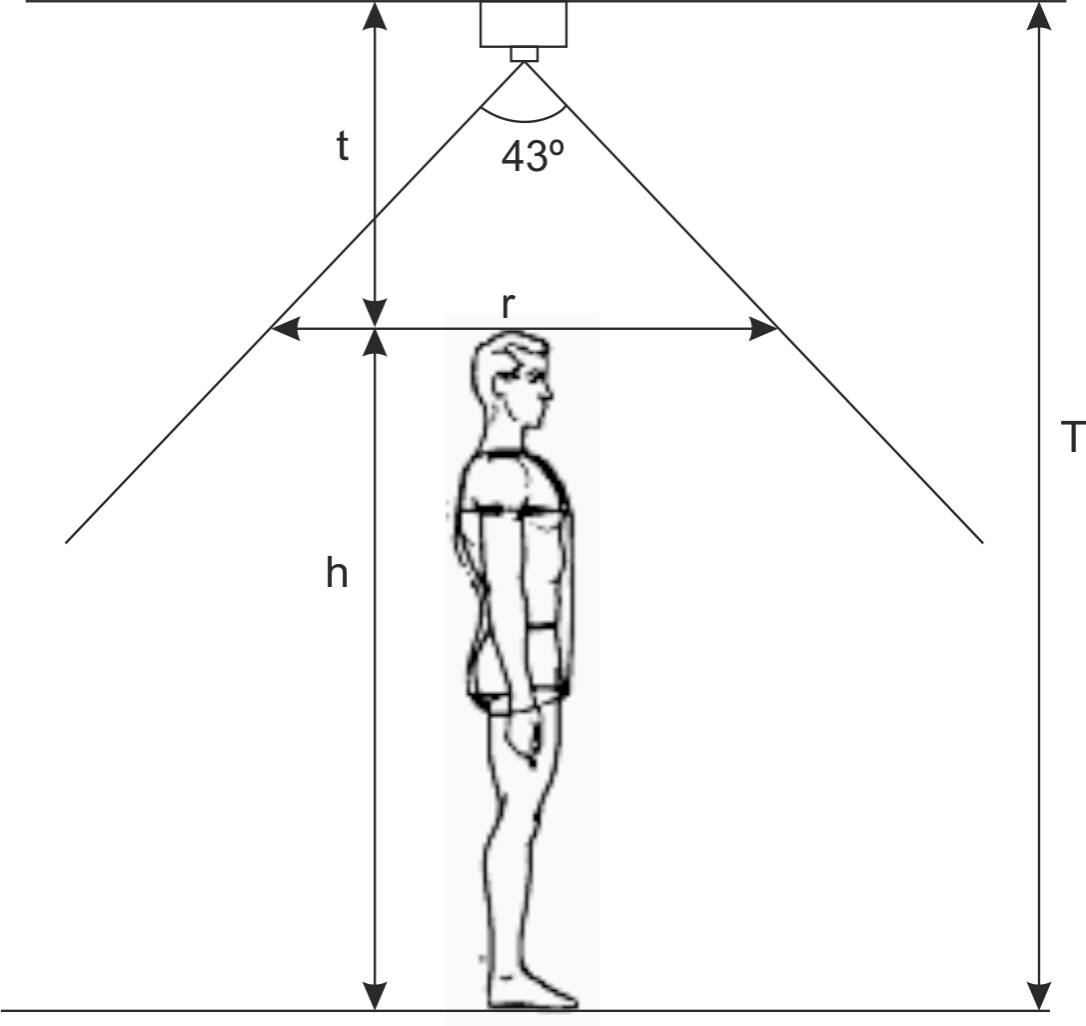
Kinect setup geometry.

Once we have argued the use of the top view configuration, we carry out a brief analysis to establish the proper camera location; see Figure 1. Since the angle of the vertical field of view of the Kinect is 43°, the maximum vertical length of the monitored area, *r*, at a height, *h*, can be computed according to the following expression:

(1) $$r=2\cdot \tan\left(\frac{43^{{}^{\circ}}}{2}\right)\left(H-h\right)$$

If we consider an average person height of *h* = 1.75*m*, fixing the position of the Kinect to *H* = 3*m* above the floor yields to *r* = 0.98*m*. For a normal walking speed of 1.4*m*/*s*, the Kinect is able to capture an average of 18 frames (considering a capture rate of 25 fps) of each person traversing the surveilled space. This number of frames must be enough to model an individual.

In this paper, we extend the preliminary study presented in [21]. Our aim is to use soft biometric features based on simple geometric features extracted from zenithal views provided by RGB-D sensors. We skip the use of the appearance information provided by the RGB cue and focus on the depth data. We claim that current consumer depth cameras can contribute to improve the identity descriptor information for the re-identification task, constituting a valuable sensor node for networked multisensor systems.

## 2. Detection

As described above, the aim of this paper is to study the possibilities of re-identifying individuals in RGB-D images acquired from a top view setup installed in an entrance door. To reduce illumination artifacts, individuals are detected and modeled based exclusively on the depth cue, using the individual trajectory information to build his/her model.

### 2.1. Background Modeling

**Figure 2.**
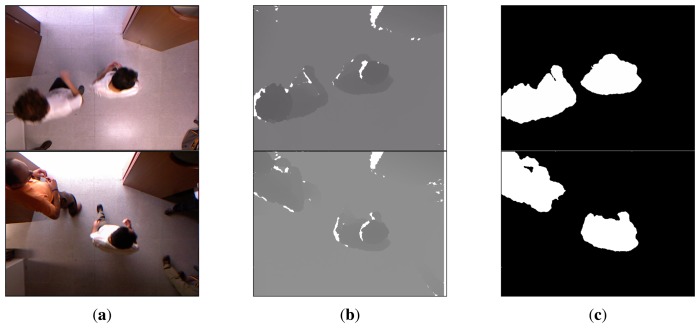
(**a**) RGBimage, (**b**) depth image and (**c**) corresponding foreground mask obtained.

Background subtraction is a common technique used to detect objects in surveillance systems. This technique requires a robust background model to be reliable. The solution is particularly simplified if the camera and lighting conditions are fixed, but the model must be robust enough to handle illumination changes. Different approaches to background modeling have been proposed, due to its inherent complexity. However, in our scenario, the use of depth information simplifies the segmentation step [14], as illumination artifacts are avoided or minimized. Additionally, since we consider the top view setup, walking people are clearly salient in the acquired depth images; see Figure 2(b).

We have adopted the background subtraction method proposed by Zivkovic and van der Heijden [22]. According to their approach, a pixel-level background model is built from a Gaussian mixture model (GMM) defined as:

(2) $$p(\vec{x}|\chi_{T},bg)\approx \sum_{m=1}^{C}{\hat{\pi }}_{m}N(\vec{x};{\hat{\vec{\mu }}}_{m},{\hat{\sigma }}_{m}^{2}I)$$

where T is the time window used to estimate the background/foreground model, *χ_T_=* {*x***^(^***^t^***^)^**,…, *x*^(^*^t^*^−^*^T^*^)^} is the training set (initial frames), ${\hat{\vec{\mu }}}_{1}$,…, ${\hat{\vec{\mu }}}_{C}$ are the mean estimations, *σ̂*_1_**,…,***σ̂_C_* are the variance estimations and *I* is the identity matrix. For each component in Equation (2), its weight is given by *π̂_m_*, so if they are sorted in descending order, the number of components *C* is obtained as:

(3) $$C=\arg\underset{b}{\min}\left(\sum_{m=1}b{\hat{\pi }}_{m}>(1-d_{f})\right)$$

where *d_f_* controls the amount of the data that can belong to foreground objects without influencing the background model. Indeed, the number of components in the GMM is not fixed as in other GMM based methods [23].

**Figure 3.**
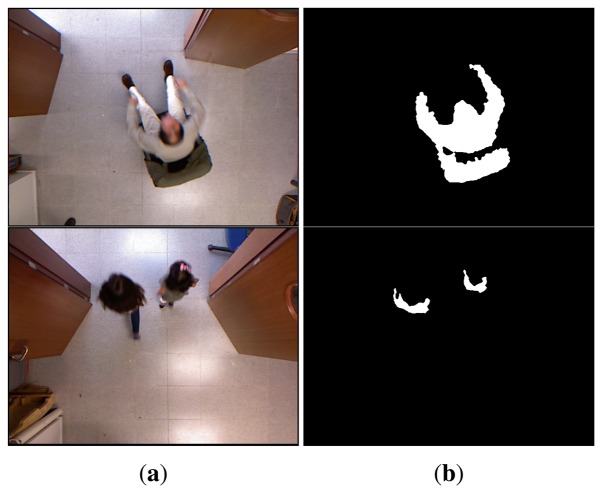
(**a**) RGB image; (**b**) corresponding foreground mask of short people.

Observing that depth images are less sensitive to shadows and illumination changes, we experimentally determined a value *d_f_* = 0.2. The reason for this is that the background model computed for the depth imagery will be much more stable than for RGB images. Given the background model in Equation (2), a pixel belongs to the foreground if the Mahalanobis distance from the pixel value to some component is less than three standard deviations. Otherwise, a new component centered in the pixel is generated. Figure 2 shows the background subtraction results for some sample frames along with their corresponding color and depth images. An advantage of this background subtraction approach is that it does not rely on any height threshold, so it can even fairly detect kids and people sitting in a chair, as can be seen in Figure 3.

Thus, according to Equation (2), a depth image pixel, *depth*(*i*, *j*), is classified as foreground using the following formula that makes use of a threshold, *c_thr_* (minimum person height):

(4) $$fg(i,j)=\{\begin{array}{ll} \text{depth}(i,j) & \text{if }p(\text{depth}(i,j)|bg)<c_{\text{trh}}\\ 0 & \text{otherwise} \end{array}$$

### 2.2. Tracking

Tracking-by-detection approaches have evidenced good performance in different unrestricted scenarios [24,25]. We have therefore adopted this focus to connect detections in terms of trajectories.

Figures 2(c) and 3(b) depict the segmentation results for different sample images based on the depth information. Large connected components in the foreground image are associated to blobs. Given the foreground image, *fg*, for frame, *L*, the set of *v* valid blobs is $B^{L}=\left\{b_{1}^{L},b_{2}^{L},\ldots ,b_{v}^{L}\right\}$. Those blobs are matched with those detected in the previous frame, $B^{L-1}=\left\{b_{1}^{L-1},b_{2}^{L-1},\ldots ,b_{m_{L-1}}^{L-1}\right\}$, by means of an overlap test. Given a blob, $b_{p}^{L}$, in the current frame, we locate the previous frame blob with larger overlap:

(5) $$mb_{p}^{L}=\arg\underset{k=1,\cdots ,m_{L-1}}{\max}(b_{p}^{L}\cap b_{k}^{L-1})$$

This test is valid in this scenario, because with people walking at normal pace, the overlap of blobs is high enough between consecutive frames. Indeed, blob tracking is simplified in this top view scenario, as occlusions are hardly ever present.

**Figure 4.**
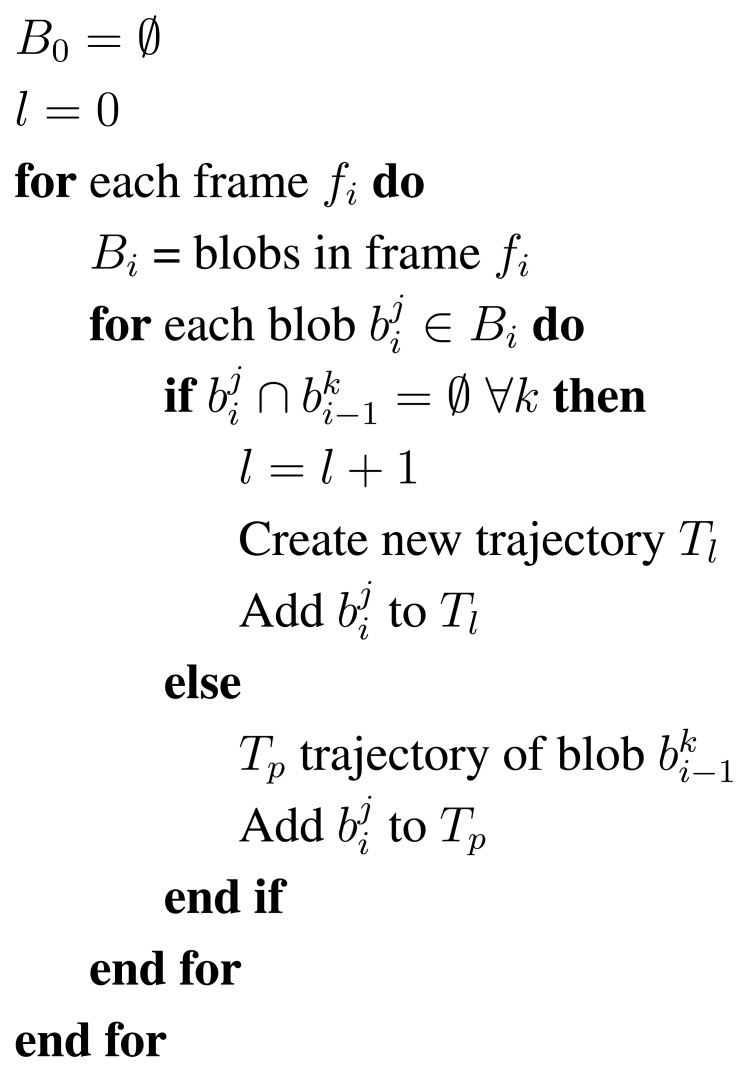
Tracking algorithm summarizing the trajectory management.

A new trajectory hypothesis is triggered each time a blob appears in the scene and no suitable matching with the previous frame is found; see the algorithm outline in Figure 4. A trajectory is then defined as a list of blobs matched and related in consecutive frames, *T_l_* = {*b_t_*_1_, *b_t_*_2_, …, *b_tl_*}, where the first trajectory blob is defined as *b_t_*_1_ and *b_tl_*, the last one. Short trajectories and those containing blobs that are too small are considered noise.

## 3. Trajectory Modeling

As defined above, given a foreground image, *fg*, the set of *v* valid blobs it contains is *B* = {*b*_1_, *b*_2_, …, *b_v_*}. In the case that a blob, *b_p_*, corresponds to a walking human, generally the closest blob pointing to the camera (lowest gray value), lies on the head; see different examples in Figure 2(b). Thus, for a given blob, its minimum is defined as:

(6) $$b_{p\min}=\min(\text{depth}(i,j);\forall fg(i,j)\in b_{p})$$

The closest point location and value are useful cues in depth images to split the blob into two parts corresponding to the head and non-head areas by a simple in-range operation similarly to Englebienne *et al.*[20]:

(7) $$\begin{array}{c} {\text{head}}_{p}\left(i,j\right)=\{\begin{array}{c} fg(i,j)\in b_{p}\wedge b_{p\min}\leq \text{depth}(i,j)\leq b_{p\min}\times {\text{thr}}_{\text{head}}\\ 0\;\text{otherwise} \end{array}\\ {\text{nohead}}_{p}\left(i,j\right)=\{\begin{array}{c} fg(i,j)\in b_{p}\wedge \text{depth}(i,j)>b_{p\min}\times {\text{thr}}_{\text{head}}\\ 0\;\text{otherwise} \end{array} \end{array}$$

**Figure 5.**
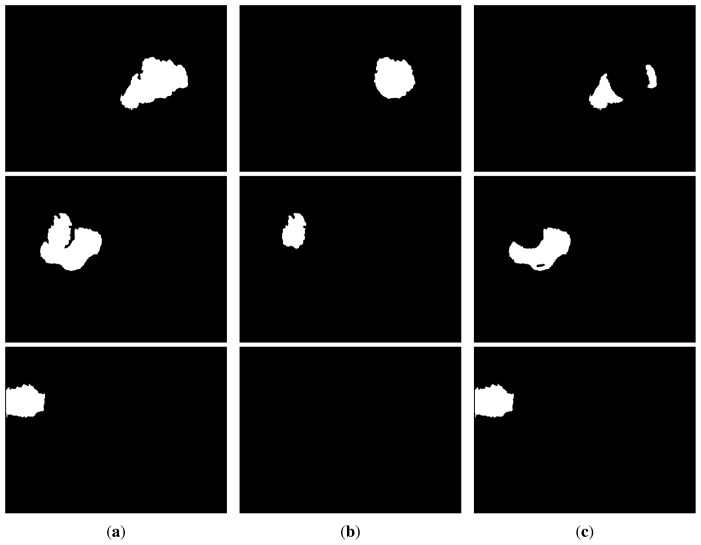
Samples of (**a**) blob, (**b**) head and (**c**) no-head areas automatically extracted.

The value of *thr_head_* is set to 1.1 according to the ideal proportions of the human body, where the head is approximately 1/8 of the body height. This process of head/no-head split is done whenever the blob container is not too close to the image border. In those situations, the head may be partially or totally out of the field of view, and the process may lead to erroneous calculations, as the highest blob point does not necessarily correspond to the head. Therefore, in situations, such as the one reflected in the third row of Figure 5, it is preferred to avoid the use of this heuristic.

This salient object detection operation applied to the sample input depth image presented in the first row of Figure 2 produces the blobs and sub-blobs shown, respectively, in the first two rows of Figure 5(b) and 5(c). As mentioned above, if a blob touches the image border, the head/no-head split is not applied, as it is shown in the third row of Figure 5. In that case, the whole blob is considered no-head or torso.

An estimation of the individual volume can be obtained using the depth of the scenario floor. To estimate the floor depth, *depth_floor_*, we assume that most of the visible area corresponds to the reference floor, *i.e.*, a plane surface. The mean depth image, $\bar{\text{depth}}$, is calculated as the average of the *k* first depth images (assuming that no individual is present) as:

(8) $$\bar{\text{depth}}(i,j)=\frac{\sum_{L=1}^{k}{\text{depth}}^{(L}(i,j)}{k}$$

where *depth*^(^*^L^*(*i*, *j*) is the pixel, (*i*, *j*), of the *L* – *th* depth image from the sequence.

On the resulting $\bar{\text{depth}}$, we calculate the mean pixel value to estimate the floor depth, *depth_floor_*, which is useful to compute the volumetric descriptors:

(9) $${\text{depth}}_{\text{floor}}=\frac{\sum_{i=1}^{\text{height}}\sum_{j=1}^{\text{width}}\bar{\text{depth}}(i,j)}{\text{width}\times \text{height}}$$

**Figure 6.**
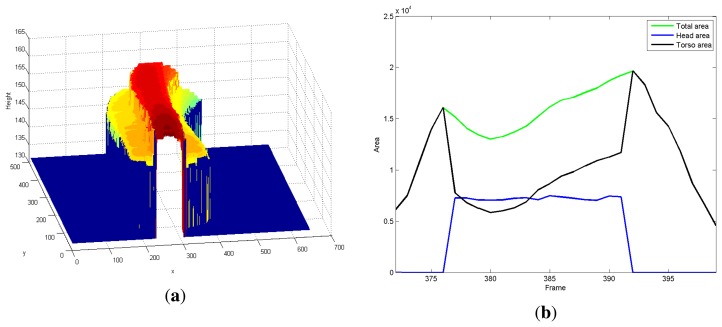
(**a**) 3D trajectory virtual volume; (**b**) area (blob and sub-blobs) related features (in pixels) extracted during a blob tracked trajectory (frames 105–120).

Figure 6(a) depicts the trajectory of a 3D virtual volume built by means of the successive combination of its tracked blobs.

After describing the blob subparts and the rough estimation of the scene floor depth, a set of features is defined. A vector of features, ${\text{vb}}_{p}^{L}$, is computed for each blob, *p*, in the current frame, *L*, including the blob area, ${\text{area}}_{p}^{L}$, the projected volume, ${\text{vol}}_{p}^{L}$, the center of the blob, $cx_{p}^{L}$, $cy_{p}^{L}$, its highest point location, $px_{p}^{L}$, $py_{p}^{L}$, and height, $b_{p\min}^{L}$. Area and projected volume features are also included for head and torso, if available.

(10) $${\text{vb}}_{p}^{L}=\left\{{\text{area}}_{p}^{L},{\text{vol}}_{p}^{L},cx_{p}^{L},cy_{p}^{L},px_{p}^{L},py_{p}^{L},b_{p\min}^{L},{\text{headArea}}_{p}^{L},{\text{headVol}}_{p}^{L},{\text{torsoArea}}_{p}^{L},{\text{torsoVol}}_{p}^{L}\right\}$$

The blob tracking described in the previous section creates trajectories in time; observing the blob descriptors presented in Equation (10), they may change over time. See, for example, the area-related features shown in Figure 6(b) for a given trajectory. Observe that the head area is not always greater than zero; indeed, its value is zero at the beginning and at the end of the trajectory This effect is due to the fact that when a person enters or leaves the scene, he/she is not completely inside the field of view. Indeed, the head and non-head split is only performed when the blob is completely inside the field of view, *i.e.*, its blob container does not “touch” the image border. To describe a trajectory, we will consider only the trajectory features computed for those frames where the head/non-head split is done; we call them the *trajectory middle life*.

A trajectory that corresponds to an individual can be characterized by a set of features extracted from the evolution of the blob features in time. Observing that during the trajectory middle life, when the head area is not zero, the trajectory features present a fairly constant behavior, we make use only of the average value of each blob feature during the trajectory middle life.

We have selected for characterization purposes the following simple and fast to compute trajectory features from Equation (10):

- **Blob height**: given by the closest to the camera blob point, which corresponds to *b_pmin_* in Equation (6).
- **Blob areas**: the blob and sub-blobs areas (head and non-head, if obtained) computed from the regions extracted according to Equation (7).
- **Blob projected volume**: the blob and sub-blobs (head and non-head, if obtained) are projected to the floor. For a blob, *b_p_*, containing *npixels* pixels, its blob projected volume is computed adding the height value of each blob pixel and subtracting the floor height, *depth_floor_*, multiplied by the number of blob pixels, *i.e., volume_bp_* =(Σ*^fg(i,j)^*^∈^*^bp^depth*(i,j)) − npixel * depth_floor_
- **Blob speed**: the mean speed in terms of pixels per second is added to the trajectory descriptor.

Thus, given a trajectory, *T_A_*, we define the set, *B_T_A__*= {*b_p_* ∈ *T_A_*}, as the blobs that make up the trajectory middle life of *T_A_*. From *B_T_A__*, the feature vector, X_A_ is:

(11) $${\text{X}}_{A}=\left({\bar{b}}_{A,}{\bar{\text{area}}}_{A},\bar{{\text{headArea}}_{A}},\bar{{\text{torsoArea}}_{A}},{\bar{\text{vol}}}_{A},{\bar{\text{headVol}}}_{A},{\bar{\text{torsoVol}}}_{A},{\bar{\text{speed}}}_{A}\right)$$

where

(12) $${\bar{b}}_{A}=\frac{1}{n_{A}}\sum_{b_{p}\in B_{T_{A}}}b_{p\min}$$

(13) $${\bar{\text{area}}}_{A}=\frac{1}{n_{A}}\sum_{b_{p}\in B_{T_{A}}}{\text{area}}_{p}$$

(14) $${\bar{\text{headArea}}}_{A}=\frac{1}{n_{A}}\sum_{b_{p}\in B_{T_{A}}}{\text{headArea}}_{p}$$

(15) $${\bar{\text{torsoArea}}}_{A}=\frac{1}{n_{A}}\sum_{b_{p}\in B_{T_{A}}}{\text{torsoArea}}_{p}$$

(16) $${\bar{\text{vol}}}_{A}=\frac{1}{n_{A}}\sum_{b_{p}\in B_{T_{A}}}{\text{vol}}_{p}$$

(18) $${\bar{\text{headVol}}}_{A}=\frac{1}{n_{A}}\sum_{b_{p}\in B_{T_{A}}}{\text{headVol}}_{p}$$

(19) $${\bar{\text{torsoVol}}}_{A}=\frac{1}{n_{A}}\sum_{b_{p}\in B_{T_{A}}}{\text{torsoVol}}_{p}$$

(20) $${\bar{\text{speed}}}_{A}=\frac{1}{n_{A}}\sum_{b_{p}\in B_{T_{A}}}\frac{\left|c_{p}-c_{p-1}\right|}{|{\text{time}}_{p}-{\text{time}}_{p-1}|}$$

being *n_A_* the cardinality of *B_T_A__*, *time_p_* the time of blob, *b_p_*, and *time_b_*_−1_ the time of the previous blob to *b_p_* in trajectory, *T_A_*. Previously, features are normalized to the range [0,1] in order to avoid bias toward features with higher ranges.

The matching of feature vectors, X*_A_* and X*_B_*, corresponding to the query subject and one subject in the gallery set, respectively, is computed as the minimum Euclidean distance between X*_A_* and X*_B_*, *d*(X*_A_*, X*_B_*):

(21) $$d({\text{X}}_{A},{\text{X}}_{B})=\underset{{\text{X}}_{B}\in \text{gallery set}}{\min}\left\{{\left\|{\text{X}}_{A}-{\text{X}}_{B}\right\|}_{2}\right\}$$

## 4. Results

To test the selected features for re-identification, we have collected data using a camera located in the upper frame of a door entrance. The resulting continuous videos have been manually annotated to get the ground truth. Below, we will describe the results for two experimental configurations: (1) *SequenceA*, containing around 14, 200 frames; and (2) *SequenceB* with 6, 000 frames. Both sequences have no restrictions imposed to the number of individuals (they respectively have around 20 and 10 different identities, all of them Caucasians and in the age range 20−)45; simultaneously present in the field of view, their speed, clothing, *etc.*

### 4.1. Trajectory Statistics

**Figure 7.**
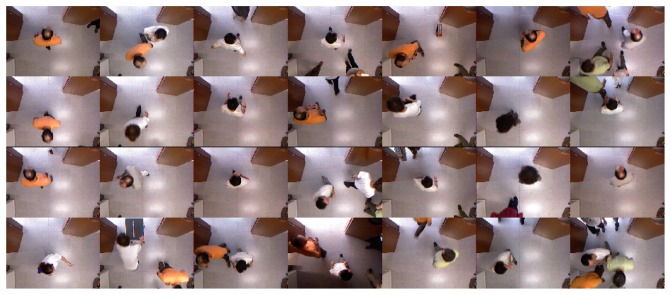
Examples of central frames of some trajectories detected as valid from SequenceA.

We have automatically removed those trajectories of individuals not completely visible during the crossing action. We define as the crossing action each time a individual crosses the monitored area under the camera. The total number of trajectories analyzed in the experiments was 211 for *SequenceA* and 54 for *SequenceB*. Figure 7 shows the central frame of some trajectories that the method tagged as valid from all the trajectories extracted in *SequenceA*. The reader may observe that there are different crossing configurations and illumination conditions.

**Figure 8.**
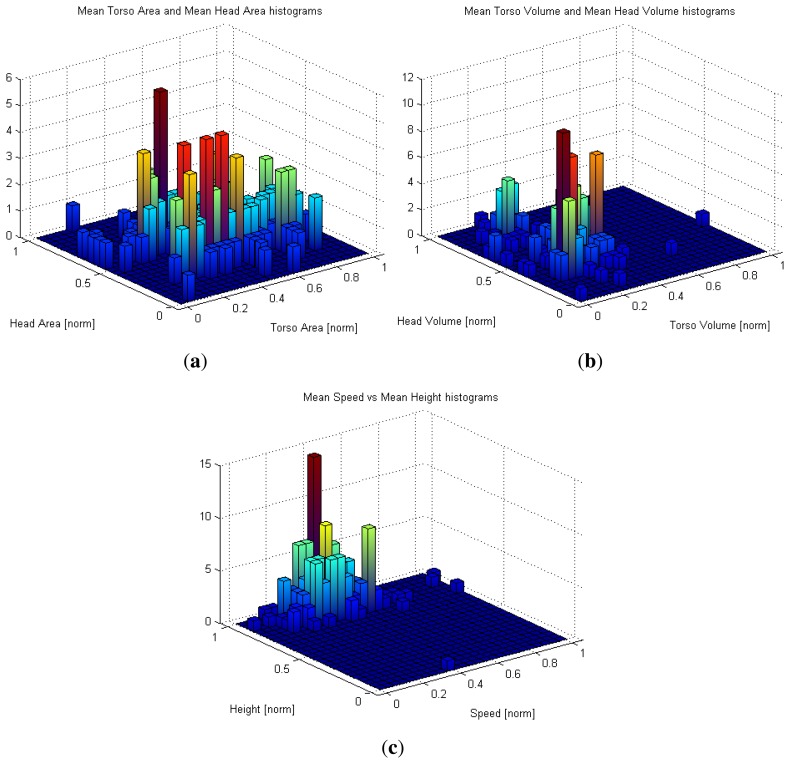
Normalized projection of (**a**) the area features; (**b**) the volume features; and (**c**) the speed and height features for the analyzed trajectories.

Histogram-based representations of the some trajectory features in *SequenceA* are presented in Figure 8. As mentioned in Section 3, each trajectory feature is computed as the mean of the values observed during the trajectory middle life, *i.e.*, when the blob could be divided into head and non-head sub-blobs. Figure 8(a,b) suggest that area and volume information are not coupled. Indeed, two blobs with the same area may project different volumes due to the height difference of the individuals to which the blobs corresponds to.

### 4.2. Re-Identification

For re-identification evaluation, we have first analyzed the longest sequence, *i.e.*, *SequenceA*. In this sequence, each trajectory is compared with the rest in a single-shot approach. This means that we have performed an experiment considering that the training set is composed by the features of a single trajectory, while the test set contains the remaining ones. Thus, the experiment is repeated 211 times for each proposed trajectory representation. Different feature vectors have been used to describe a trajectory:

- **AH**: only the area (head and torso) and height features of Equation (10) are used.
- **AHV**: the area (head and torso), height and volume (head and torso) features of Equation (10) are employed.
- **AHVS**: the area (head and torso), height, volume (head and torso) and speed features of Equation (10) are employed.

**Figure 9.**
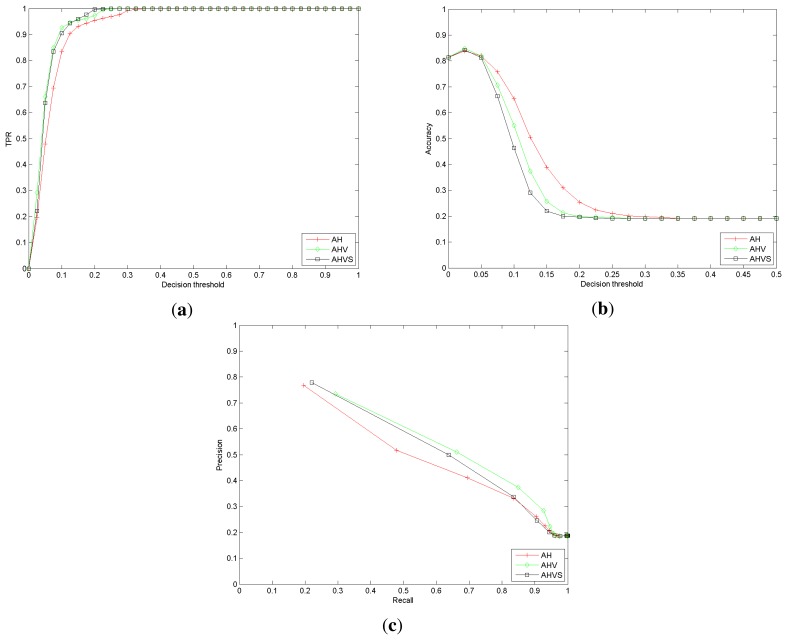
(**a**) Recall or true positive rate (TPR); (**b**) accuracy; (**c**) precision *vs.* recall.

For each re-identification, the decision threshold defines if the re-identification is correct or not attending to the distance. The performance evaluation is done using recall, accuracy and precision. The receiver operating characteristic (ROC) curve is computed for the nearest neighbor (NN) classifier, considering different decision threshold values. The summarized results are depicted in Figure 9.

As expected, raising the decision threshold increases the recall or true positive rate (TPR), but reduces, almost simultaneously, the accuracy. The use of more features to describe the trajectory seems to improve the recognition rates. Nevertheless, the inclusion of the speed feature (*AHV S* variant) does not introduce any discriminant information; indeed, the performance decreases. Certainly, if an individual modifies his speed in different observations, the descriptor is not valid to re-identify him/her. However, as described below, speed can be used for detecting unexpected situations.

The results for *SequenceA* indicate that, apparently, a set of simple features provides useful information to re-identify individuals. We can conclude that even using such a set of naive and weak descriptors, the individual re-identification performances are promising. Focusing, for instance, on Figure 9(c), if the decision threshold is set to 0.05, the precision is close to 50% and the recall to 64%. Observe that no appearance-based descriptor has been used in the experiments.

**Figure 10.**
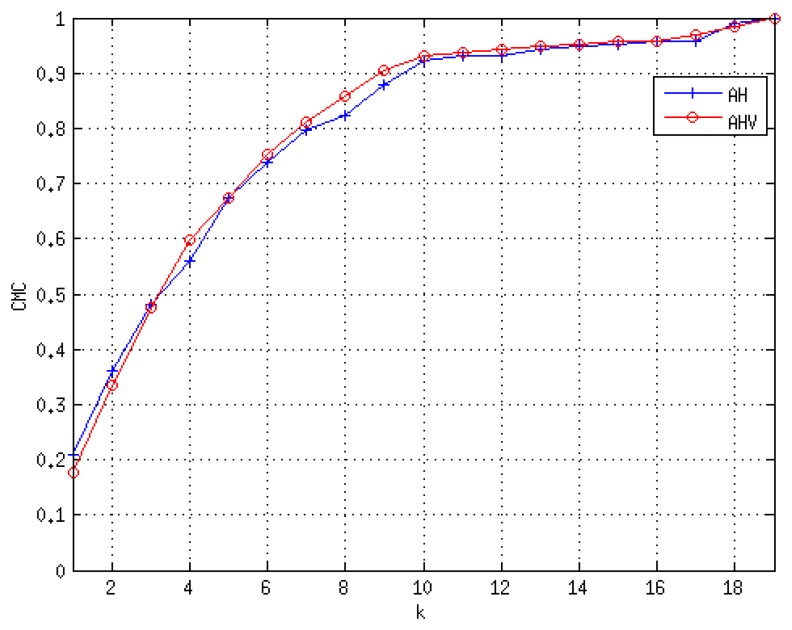
Cumulative Matching Curve for *SequenceA*.

**Figure 11.**
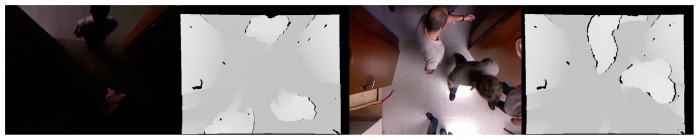
RGB and depth shots exhibiting hard illumination conditions.

**Figure 12.**
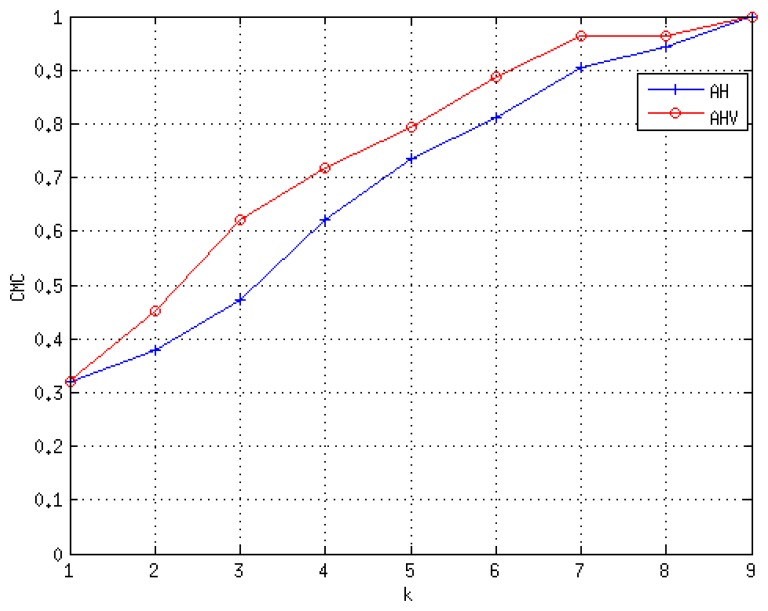
Cumulative Matching Curve for *SequenceB*.

To provide a better understanding for the re-identification problem, we include the Cumulative Matching Characteristic (CMC) curve for both sequences, but only considering approaches *AH* and *AHV*, *i.e.*, eliminating the speed-based feature. The CMC curve provides the probability of finding the true identity among the first *k* models. For *SequenceA*, the CMC curve is shown in Figure 10. The integration of more features in the model seems to improve the identity discrimination. In *SequenceB*, we have imposed the condition to have a similar number of crossings per individual. The total number of individuals is nine, and the total number of analyzed crossing actions was 54. As depicted in Figure 11, this sequence presents hard illumination conditions, as there are severe illumination changes. During the first half of the sequence, the lights are off and then switched on. Indeed, in the first part of the experiment, appearance-based approaches would not be able to detect, track and model the different identities, due to the semi-darkness conditions. However, the geometric-based model proposed may solve those situations to some extent, as is suggested in the CMC curve of this sequence, see Figure 12.

### 4.3. Unexpected Features Detection

In this subsection, we present the results of exploring the use of the features defined in Equation (10) to detect unusual objects or behaviors.

**Figure 13.**
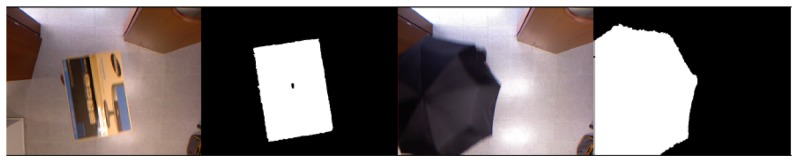
Two samples of selected situations with too large detected heads (left) and blob (right) size.

In Figure 8(a), we plot the head blob and no-head blob normalized mean area for each trajectory in the sequence. Our first aim is to detect trajectories associated to blobs whose dimensions correspond to outliers and unexpected dimensions, a circumstance that could suggest the presence of unexpected object/behavior in the monitored area. Indeed, the plot indicates the presence of area values quite different to the mean. Those peaks correspond to situations similar to those presented in Figure 13. The blob or head blob dimensions are abnormal; therefore, an event can be triggered to assist a potential human operator. If the system task is devoted to people counting, such situations can evidence the possible intentionality of someone to hide himself from the automatic surveillance system. During the experiments, the system was able to detect all trajectories belonging to unusually big blobs (0.5% of the total). In Figure 13, two examples of unusually big object detections are shown.

**Figure 14.**
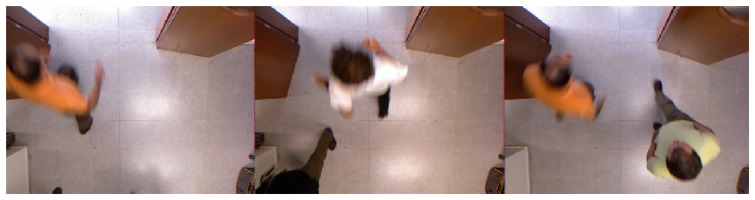
Trajectory middle frame of those labeled as very fast.

Speed is another observable trajectory feature that has no discriminative power according to the above results in re-identification, but it can alert about abnormal behaviors. Their normalized distribution is depicted in Figure 8(c). Observing the average trajectory speed, there are some trajectories suggesting a rather faster or slower behavior. By comparison of a trajectory mean speed value with the overall average speed, it is possible to label a trajectory as very slow, slow, average, fast or very fast. In an ambient intelligence scenario for elderly people, an abnormally slow speed can be considered as a cue of a health problem and trigger an alarm. Figure 14 presents the central frame of those trajectories labeled as very fast. They correspond to running individuals, as suggested by the present blur. Observing the shift of blobs during a given interval, it is also possible to detect if someone has stopped or slowed down in the monitored area for a while. Depth information acquired from the top view is therefore useful to detect those behaviors. During the experiments, the system was able to detect all trajectories associated to running individuals (1% of the total analyzed trajectories) and presenting a stop event for more than 10 frames.

## 5. Conclusions

We have made use exclusively of the depth information provided by a consumer RGB-D camera to detect, track and describe individuals crossing a monitored area. The selected top view configuration preserves privacy and eases the task, making it simple to extract different trajectory features. Also, this setup introduces robustness, due to the lack of occlusions among individuals.

No appearance information is collected to model the individuals, just simple geometric descriptors extracted from the depth image blob. Their discriminative power has provided promising results in the set of experiments performed under severe changes in illumination, where appearance information, such as color, cannot be gathered.

The set of geometric features has been selected attending to its computational cost. This low computational cost makes the development of standalone systems based on embedded architectures affordable. An experimental setup has been carried out in an entrance door scenario, where two sequences summing more than 20, 000 frames and 300 crossing events under illumination changes have been manually annotated. In both sequences, the proposal has been able to re-identify the individuals with a fair accuracy.

The system can additionally be integrated as a source of high semantic level information in a networked ambient intelligence scenario, to provide cues for different problems, such as detecting abnormal speed and dimension outliers, that can alert of a possible uncontrolled circumstance.

## Supplementary Files

Supplementary File 1 Note (PDF, 4 KB)
