# Supplementary material for: On the Use of Simple Geometric Descriptors Provided by RGB-D Sensors for Re-Identification
Source: Sensors (Basel). 2013 Jun 27;13(7):8222–38. doi: 10.3390/s130708222 (PMC3758592; doi:10.3390/s130708222)
Supplement: Supplementary File 1 — Note (PDF, 4 KB) [file sensors-13-08222-s001.pdf]

Note:

On page 8230, Equation (17) appears as an empty line. This is a print error and nothing is missing.
